# The Irie Classroom Toolbox: Mixed method assessment to inform future implementation and scale-up of an early childhood, teacher-training, violence-prevention programme

**DOI:** 10.3389/fpubh.2022.1040952

**Published:** 2022-12-13

**Authors:** Marsha Bowers, Taja Francis, Helen Baker-Henningham

**Affiliations:** ^1^Caribbean Institute for Health Research, University of the West Indies, Kingston, Jamaica; ^2^School of Human and Behavioural Sciences, Bangor University, Bangor, United Kingdom

**Keywords:** violence prevention, teacher-training, violence against children, early childhood, preschool, behaviour change, low-and middle-income country

## Abstract

**Introduction:**

Violence against children (VAC) is a violation of child rights, has high prevalence in low- and middle-income countries, is associated with long-term negative effects on child functioning, and with high economic and social costs. Ending VAC at home and at school is thus a global public health priority.

**Methods:**

In Jamaica, we evaluated an early childhood, teacher-training, violence-prevention programme, (the Irie Classroom Toolbox), in a cluster-randomised trial in 76 preschools. The programme led to large reductions to teachers' use of VAC, although the majority of teachers continued to use VAC at times. In this paper, we describe a mixed-method evaluation of the Irie Classroom Toolbox in the 38 Jamaican preschools that were assigned to the wait-list control group of the trial. In a quantitative evaluation, 108 preschool teachers in 38 preschools were evaluated at pre-test and 91 teachers from 37 preschools were evaluated at post-test. One preschool teacher from each of these 37 preschools were randomly selected to participate in an in-depth interview as part of the qualitative evaluation.

**Results:**

Preschool teachers were observed to use 83% fewer instances of VAC across one school day after participating in the programme, although 68% were observed to use VAC at least once across two days. The qualitative evaluation confirmed these findings with all teachers reporting reduced use of violence, but 70% reporting continued use of VAC at times. Teachers reported that the behaviour change techniques used to deliver the intervention increased their motivation, knowledge and skills which in turn led to improved child behaviour, improved relationships and improved professional well-being. Direct pathways to reduced use of VAC by teachers were through improved child behaviour and teacher well-being. The main reasons for continued use of VAC were due to barriers teachers faced using positive discipline techniques, teachers' negative affect, and child behaviours that teachers perceived to be severe.

**Discussion:**

We describe how we used the results from the mixed-method evaluation to inform revisions to the programme to further reduce teachers' use of VAC and to inform the processes of training, supervision and ongoing monitoring as the programme is scaled-up through government services.

## Introduction

Violence against children (VAC) is a global public health problem with high prevalence in low- and middle-income countries (LMIC). Two thirds of children aged 2–4 years living in LMIC, equivalent to more than 220 million children, experience physical punishment or psychological aggression at home (1, 2). Over half a billion children each year experience violence in and around schools, including VAC by teachers (3). Article 19 of the Convention of the Rights of the Child states that children have the right to be protected from “all forms of physical and mental violence” and VAC is a clear violation of children's rights. VAC has long-term negative effects on children's physical and mental health and academic achievement and increases the risk for later perpetration of child and spousal abuse thus leading to an intergenerational cycle of violence (1, 2, 4–6). There are also large economic costs associated with VAC. In a 2014 report, global costs of VAC were estimated to be $7 trillion (between 3 and 8% of global GDP) (7), while a more recent report on ending violence in schools estimated that school violence alone costs US$11 trillion in lost future earnings caused by children learning less while in school and dropping out of school (3).

With the need to protect child rights, the high prevalence, the long-term negative effects on child functioning, and the high economic costs of VAC, ending violence against children is recognised as a global public health priority. Eliminating violence against children is included in the Sustainable Development Goals with goal 16.2 calling for an end to all forms of violence against children (8). The Global Partnership to End Violence against Children was created in 2016 to address this goal (https://www.end-violence.org) and the World Health Organisation launched the INSPIRE framework which includes seven evidence-based strategies for ending VAC (9). One of these strategies involves implementing parent and caregiver support programmes.

There is growing evidence from LMIC that violence prevention, parenting programmes can be effective in reducing parents' use of VAC at home (10, 11), although most studies have been small efficacy trials and evidence is needed on their effectiveness at scale. There is some limited evidence of the effectiveness of interventions to prevent VAC by primary and secondary school teachers (12–14), but less work has been conducted in early childhood educational settings such as preschools and childcare centers. Ending teachers' use of VAC is critical as schools reach large numbers of children and children spend a large amount of time there. The mission of schools is to promote children's learning, social-emotional competence, wellbeing, and life skills. VAC by teachers leads to school drop-out, poor health and wellbeing, physical injury, and low levels of learning (3). Interventions to prevent violence at school can thus support schools to achieve their mission of providing quality education. Early childhood is a particularly sensitive period for children's development and safe, secure, stimulating and nurturing early childhood caregiving environments promote children's cognitive and behavioural functioning and their longer-term health and development (15).

In Jamaica, violence against young children is common at school and at home with 84% of parents of children aged two-to-four years reporting using physical punishment over the past month (16) and 88% of preschool teachers observed to use VAC over two school days (17). There is thus an urgent need for violence-prevention programming during the early childhood years. This need has been recognised at the national level – Jamaica is a pathfinder country in the Global Partnership to End Violence Against Children and the government has launched ‘The National Plan of Action for an Integrated Response to Children and Violence' (18). To respond to the need for violence-prevention programming, we have developed, implemented, and evaluated two early childhood programmes to reduce VAC at school and at home: (1) the Irie Classroom Toolbox: a teacher-training programme (17, 19), and (2) the Irie Homes Toolbox: a parenting programme (20, 21).

During the initial development, implementation, and evaluation of The Irie Toolbox programs, we have utilised key implementation science principles to increase the likelihood that the program will be integrated into the existing early childhood educational system in a sustainable way and maintain effectiveness at scale. These principles include designing the interventions for scale from the outset (19, 20), and embedding monitoring and evaluation activities, (including quantitative, qualitative and process evaluations), into ongoing programme implementation with lessons learnt used to inform revisions to the intervention (20, 22–24). We have given an overview of the processes involved in designing, implementing, evaluating, and initial scaling of the Irie Toolbox programmes in a recent article (Baker-Henningham et al.)^1^. The processes used include principles of the measurement for change approach that advocates using a monitoring, evaluation and learning (MEL) system guided by five interconnected concepts (25). In the Measurement for Change approach, MEL systems strive to be: (1) dynamic (flexible and responsive), (2) inclusive (involving all stakeholders), (3) informative (collecting and utilising information from a variety of sources), (4) interactive (measuring interactions among participants), and (5) people centered (acknowledging and measuring individual differences). Through utilising this framework, ECD researchers and practitioners can ensure that their MEL systems are used to adapt and iteratively revise interventions to meet changing needs as they are scaled up.

This article demonstrates how we used the Measurement for Change approach by embedding MEL activities into one round of implementation of the Irie Classroom Toolbox. At the time of this study, we had previously evaluated the Irie Classroom Toolbox in a cluster randomised controlled trial in seventy-six preschools and demonstrated that the Toolbox led to large reductions in teachers' use of VAC and significant improvements to the quality of the classroom environment, class-wide child prosocial behaviour and teacher wellbeing (17). However, the majority of teachers continued to use VAC at times indicating the need to further strengthen the intervention. The data for this study was collected when the Irie Classroom Toolbox was implemented with teachers in the preschools originally assigned to the wait-list control group of the trial. The aim was to collect quantitative and qualitative data that would inform future implementation of the Irie Classroom Toolbox including: 1) revisions to the content of the intervention to strengthen effectiveness in reducing VAC by teachers, 2) the design of the training and supervision processes as the programme is scaled-up through government services, and 3) the development of monitoring tools required to promote quality implementation. We conducted a pre-post quantitative evaluation of the effect of the intervention on teachers' use of violence against children, the quality of the classroom environment, class-wide child behaviour and teacher depressive symptoms to evaluate the benefits of the Toolbox training with this cohort of teachers. We also conducted a qualitative evaluation of the intervention to investigate teachers' perceptions of the mechanisms of action of the intervention.

## Methods

### Study design and participants

This mixed-method study was conducted with the wait-list control arm of a cluster-randomised trial in community preschools in Kingston and St. Andrew, Jamaica. Community preschools cater to children aged 3–6 years and are run by community organisations with government support and oversight. In the original trial, 76 preschools were randomly selected from 120 eligible preschools to participate in the study. Preschools were eligible to participate if they had two-to-four classes of children, a minimum of ten children in each class and were located in urban areas of Kingston and St Andrew. All teachers and all classrooms in selected preschools participated in the study. Preschools were randomly assigned to receive the Irie Classroom Toolbox program (*n* = 38) or to a waitlist control group (*n* = 38). Measurements were conducted at baseline (May–June, 2015), post-test (May–June, 2016), and one-year follow-up (May–June, 2017) in all 76 preschools. The results of this trial have been published previously (17).

From August 2017 to April 2018, teachers in the preschools allocated to the waitlist control group participated in the Irie Classroom Toolbox training program. All teachers in the sample preschools participated in the study. Measurements conducted from May–June, 2017 in these 38 schools (the one-year follow-up point of the original trial) were used as the pre-test measurements in this study. Post-test measurements were conducted from May–June 2018. For the qualitative evaluation, we randomly selected one teacher from each preschool to participate in an in-depth, semi-structured interview. No teachers refused to participate. Interviews were conducted in June 2018.

Ethical approval for the study was given by the School of Psychology, Bangor University ethics committee (2014-14167) and the University of the West Indies ethics committee (ECP 50, 14/15). Written, informed consent was obtained from the preschool principal and all preschool teachers in each school to participate in the study. Separate written informed consent was obtained for teachers selected to participate in the in-depth interviews.

### Intervention

Teachers were trained in the Irie Classroom Toolbox through four full-day teacher training workshops, eight one-hour sessions of in-class support (once a month for 8 months) and fortnightly text messages. The Irie Classroom Toolbox trains teachers in classroom behaviour management and how to promote children's social and emotional skills (20). See Table 1 for full details of the intervention.

**Table 1:** The Irie Classroom Toolbox intervention.

**Content** The Irie Classroom Toolbox includes four modules: (1) Creating an emotionally supportive classroom environment, (2) Preventing and managing child behaviour problems, (3) Promoting children's social and emotional competence, and (4) Individual and class-wide behaviour planning. **Procedures** The content was introduced through four 6-hour teacher-training workshops, eight 1-hour in-class individual support sessions (once a month for eight months) and fortnightly text messages over an eight month period (from late August-late April). Teachers were also given monthly classroom assignments. Workshops Two workshops were held in the summer holiday prior to the start of the new school year (late-August), one workshop was held in the Autumn half-term and the final workshop in the Spring half-term. Teachers were split into four groups and each workshop was conducted by a facilitator and a co-facilitator with groups of 20-30 participants. During workshops, teachers were introduced to the content through demonstration, live and video modeling, role-play and rehearsal, group discussions, and small group activities. Workshops were designed to be practical, participatory and fun with an emphasis on supporting and motivating teachers through the use of scaffolding, collaborative problem-solving, reflecting listening and positive feedback. In-class support Each in-class support session was designed to support teachers with a specific topic covered in workshops. The topics included: (1) Using praise, (2) Teaching classroom rules, (3) Coaching children's academic skills, behaviour, friendship and emotions, (4) Interactive reading, (5) Explicitly teaching friendship skills, (6) Problem-solving stories, (7) Preventing problems in the classroom, (8) Review. The in-class support consisted of three discrete elements: (1) Planning: the coach and teacher discussed the aims of the session (5 mins), (2) Coaching (45 mins): the coach worked alongside the teacher modeling how to use the strategies, encouraging the teacher to use the strategies and giving positive feedback, and highlighting the effects of the teacher's use of the strategies on the children, and (3) Debriefing (10 mins): The coach and teacher evaluated the session, and engaged in collaborative problem-solving and goal setting. Text messages Fortnightly text messages were sent to all teachers. The messages related to the content covered during the monthly coaching sessions and were designed to remind and motivate teachers to use the strategies. Classroom assignments Classroom assignments involved practical activities that encouraged teachers to use the strategies covered in the coaching session during the next month and record the effect of the strategies on the classroom environment, and on children's engagement and behaviour. Teachers were given classroom assignments after the first seven in-class support sessions. **Materials** The Irie Classroom Toolbox includes resources for teachers and resources for facilitators. Resources for teachers ***IRIE Classroom Tools Book***: Describes each strategy, how and why to use it. ***IRIE Classroom Activities Book***: Lesson plans to teach classroom rules, friendship skills and emotions, songs, games to build children's self-regulation skills, activities to reinforce friendship skills, activities to reinforce children's knowledge of emotions. ***IRIE Classroom Resource Book:*** Behaviour planning forms, Irie Notes (to share positive news with parents). ***Three sets or picture cards***: Rules, Friendship Skills and Emotions. ***Problem-solving Stories Book***: 14 pictorial stories of common problems children face at school. Resources for facilitators ***IRIE Classroom Teacher-Training Kit***: A kit containing materials required to conduct the training including a fully-scripted training manual, video vignettes, charts, cards for sorting, and selected toys and picture books. ***IRIE Classroom Coaching Manual***: Guidelines on how to conduct the in-class support sessions. ***IRIE Classroom Monitoring Tools***: Self-evaluation and teacher-evaluation forms, observation checklists to monitor teachers' use of the strategies in the classroom. **Staffing** The intervention was implemented by eight female staff hired and trained by the research team. All staff had Masters' degrees and had delivered the Irie Toolbox intervention in the first round of implementation in the 2015-2016 school year. Four staff facilitated the teacher-training workshops and four staff co-facilitated the workshops. The co-facilitators also coached the teachers during the in-class support sessions and each coach was responsible for 9-10 preschools (25-30 teachers). During the first round of implementation, facilitators and co-facilitators received twelve days of training in how to conduct the teacher-training workshops and attended weekly group training and supervision meetings over the eight months of implementation to practice the skills required for the in-class support sessions and to problem-solve issues as they arose. Coaches also received monthly field supervision from two of the workshop facilitators. In this second round of implementation, staff participated in four days refresher training in how to conduct the teacher-training workshops (1 day of training prior to each workshop) and participated in weekly group supervision meetings throughout the eight months of implementation. Coaches continued to receive monthly field supervision. **Implementation** Teacher satisfaction Teacher satisfaction with workshops was measured using evaluation forms that were completed after each workshop in which teachers rated the content, videos, facilitator skills, group discussions, demonstrations and small group discussions on a six-point scale (0=not at all helpful to 5=extremely helpful). Teacher satisfaction was high with a mean (SD) score across all four workshops of 26.7 (2.6) (out of a maximum of 30). Teacher participation and engagement Teachers attended a mean (SD) of 2.9 (1.1) workshops (out of a maximum of four) and participated in a mean (SD) of 7.7 (0.8) in-class support sessions (out of a maximum of eight). Twenty-nine teachers (31.9%) attended all four workshops, 78 (85.7%) attended at least two workshops, 1 (1.1%) attended zero workshops. Seventy-nine teachers (86.8%) participated in all eight in-class support sessions, eighty-three (91.2%) participated in seven or more and all participated in at least four. Teachers completed a mean (SD) of 3.8 (2.7) classroom assignments (out of a maximum of seven). Seventy-eight teachers (85.7%) did at least one assignment, forty-nine teachers (53.8%) did four or more.

We had previously implemented the Irie Classroom Toolbox over a full school year with teachers in the 38 schools assigned to the intervention group in the cluster-randomised trial. During this year, workshop facilitators and in-class coaches received ongoing supervision and support and ongoing revisions were made to the teacher-training and in-class support manuals to address problems and needs as they arose. Hence during this second round of implementation, the staff were more experienced and confident and the facilitator manuals were more comprehensive. The content and process of implementation of the Toolbox and the materials given to the teachers remained largely unchanged.

### Measurements

Measurements included quantitative and qualitative data. All quantitative measurements have been used previously in early childhood classrooms in Jamaica (17, 22).

#### Quantitative data

The primary outcome was teachers' use of violence against children measured through independent structured observations throughout one school day. Event sampling was used to record each discrete act of violence against children including teachers' use of: (1) physical violence (e.g., hitting with hand or object, pinching, poking, forcefully pushing or pulling a child, making a child stand or kneel in uncomfortable positions), and (2) psychological aggression (e.g., name-calling, threatening physical punishment, encouraging children to hit or harm each other, using non-verbal threats). The total score was the number of times the teacher used violence against children throughout the day (see Supplementary material). All behaviours were defined in an observation manual with clear definitions of each behaviour, examples and non-examples, and decision rules.

Secondary outcomes included observations of the classroom environment, a binary measure of observed teachers' use of violence across two schools days, and teacher depressive symptoms. The observations of the classroom environment included: (1) two measures of the quality of the classroom environment assessed using the Classroom Assessment Scoring System Pre-K (CLASS Pre-K): emotional support and classroom organisation (26), and (2) two measures of class-wide child behaviour: class-wide child aggression and class-wide child prosocial behaviour assessed using rating scales that measured the frequency, intensity and number of children involved in aggressive and prosocial behaviour respectively. These classroom observations were conducted over five 20-minute periods over one school day with the mean score over the five observations used in the analyses. They were scored on a seven-point rating scale (1–7) where 1 = low and 7 = high. During these five 20-minute observation periods, the observers also recorded whether teachers used violence against children (including physical punishment and psychological aggression) and a binary score of teachers' use of violence over two school days was created. Teacher depressive symptoms was measured by interviewer-administered questionnaire using the Centre for Epidemiological Studies Depression Scale (27).

##### Procedure and quality control

Data was collected by a team that included ten teacher observers who conducted observations of teachers' use of violence across one school day, ten classroom observers who conducted observations of the classroom environment over another school day and one teacher interviewer. To prevent bias, the observers and teacher interviewer were unaware that the teachers had participated in an intervention. Teachers were not masked. Each classroom was observed for five 20-minute periods over one school day by a classroom observer and then on a second day, a teacher observer conducted observations of teachers' use of violence across the whole school day. When all observations in the preschool were completed, the teacher interviewer visited the school to conduct teacher interviews. Only one observer was present in a classroom at a time and a maximum of two observers were present in a preschool each day.

Training for observers was conducted over a 4 week period at each measurement point and included 1 week of in-office training, 2 weeks of field training and 1 week to conduct interobserver reliabilities prior to the start of data collection. Interobserver reliabilities were measured using intraclass correlation coefficient (ICC) and were >0.8 for all classroom observations and > 0.9 for observations of teachers' use of violence over one school day. We conducted ongoing reliabilities once a week with each observer throughout each data collection period and interobserver reliabilities of ICC > 0.8 for observations of the classroom environment and ICC > 0.9 for teachers' use of violence were maintained.

The measure of teachers' depressive symptoms (CES-D) had high internal consistency (Cronbach's alpha= 0.88 at pre-test, 0.89 at post-test) and high test-retest over a 2 week period (ICC = 0.82). For the observational measures, we previously calculated stability over 1 year using data from the wait-list control group (17). Stability was in the expected range for all outcomes except class-wide prosocial behaviour which showed low stability: teachers use of violence against children, ICC = 0.63; emotional support, ICC = 0.48; classroom organisation, ICC = 0.42; class-wide aggression, ICC = 0.59; class-wide prosocial behaviour, ICC = 0.13; violence against children over two school days, ICC = 0.51.

#### Qualitative data

In-depth semi-structured interviews with each teacher selected to participate in the qualitative evaluation were conducted by one of two research assistants who had not worked with these teachers previously. Both research assistants were female with Masters' degrees in Psychology. They received 10 days' training in conducting the in-depth interviews including theory, demonstration, role play and supervised practice interviewing teachers. The interview focussed on how the intervention led to changes in teachers' use of VAC and/or reasons for teachers' continued use of VAC. The interview guide was developed by MB and HBH and piloted by MB, HBH and the two interviewers with four teachers who were not selected to participate in the in-depth interviews. The interview guide is shown in Table 2. Interviews were conducted in a quiet area on the school property, after all post-test measurements in the school were completed, and were scheduled in advance at a time convenient for the teachers. Each interview lasted between 45 and 90 min and was audio-recorded and then transcribed. Transcriptions were independently checked for accuracy against the audiotape. Each teacher was given an identification number to preserve their anonymity. Children's, teachers' and schools' names were excluded from the transcriptions.

**Table 2:** Interview guide for in-depth semi-structured interviews.

**Topic**	**Questions with suggested prompts**
**Opening questions**	Tell me what you thought about the training programme that you participated in. What are you doing differently because of the training programme?
**Corporal punishment**	Some teachers tell us that they sometimes need to give children a little slap, threaten to slap them or shout at them to get them to behave. To what extent do you find that? Under what circumstances/for what behaviours do you find you need to ‘give a little slap' or shout at children? *(If the teachers report not using violence against children (VAC) or report a reduction in use)* Suggested prompts What led to the difference? What do you do instead? What strategies are most helpful? What strategies do you use the most? What made it easier for you to use those strategies? How do those strategies help? *[If teachers report that they use corporal punishment (even if reduced)]* Suggested prompts One of the aims of our training programme is to reduce teachers' use of *(use the teacher's words to describe VAC)*. What are some of the reasons why you need to use *(use the teacher's words to describe VAC)*? Under what circumstances/for what behaviours do you find you need to *(use the teacher's words to describe VAC)?* What would you need to help you to manage children's behaviour without needing to *(use the teacher's words to describe VAC)?*
**Strategies used the least**	What strategies do you use the least? What made it harder to use these strategies? What would need to be different to increase your use of these strategies? Are there any strategies which you do not agree with?
**Teacher training workshops**	*(If the teacher attended at least one workshop)* What did you think about the workshop(s)? What activities were most helpful? How did the activities help you? What was less helpful in the workshop?
**In-class support**	What did you think about the in-class support? What did you like about it? What did you dislike? What effect did it have on you using the strategies?
**Summary**	If you were asked to share one strategy with a teacher who had not received the training, which strategy would you share? Why did you choose that strategy? Do you have any advice on how the programme can be improved for other teachers who participate at a later date?

### Analysis

#### Quantitative analysis

The difference between pre-test and post-test scores was analysed using a Wicoxon Paired Signed Rank Test for teachers' use of violence over one school day, a paired *t*-test for all other continuous variables, and a Chi-Squared test for the binary variable of teachers' use of violence over two school days. Teachers' depressive symptoms was normalised using a square root transformation prior to conducting the paired *t*-test.

#### Qualitative analysis

Qualitative Data was analysed manually using the framework approach which is appropriate for applied policy research that has specific objectives and is based on a priori issues (28). The framework approach involves five steps: (1) reading and rereading the transcripts in a familiarisation phase, (2) constructing an index of codes based on the themes and subthemes in the data, (3) applying the codes to the transcripts, (4) creating tables to collate all codes related to each theme and subtheme, and 5) interpreting the data. Steps one and two were conducted by MB and HBH who read six transcripts and collaborated on developing the coding index using inductive and deductive methods. Initial codes were generated using the interview guide and inductive codes were added as new themes and subthemes emerged from the data. In step three, all text was coded and where a section of text included more than one code, all relevant codes were applied. In step four, the data was reorganised into tables of each theme/subtheme and we report the number of participants who mentioned each subtheme as an indication of its salience within the data. In step five, we examined the data and constructed mechanism diagrams to represent teacher reports of the pathways to their reduced use of VAC and continued use of VAC. Data was coded by MB in discussion with HBH with regular meetings to address any queries. In addition, another member of the Irie Toolbox team independently coded six teacher transcripts and we found acceptable levels of agreement (>80% on all codes). Discrepancies were resolved through discussion with HBH and MB.

## Results

### Quantitative evaluation

In May-June, 2017, data was collected with 108 teachers in 38 preschools. In May-June, 2018, post-test data was collected with 91 teachers in 37 preschools: a loss of one preschool and seventeen teachers. Fourteen teachers had left the school and three teachers were principals who were no longer responsible for teaching a class. There were no significant differences between those found and lost on classroom or teacher characteristics or on pre-test scores of the outcome variables (see Supplementary Table 1).

Teacher and classroom characteristics are shown in Table 3. At pre-test, teachers used a median [interquartile range (IQR)] of 6 (1–18) instances of violence over one school day and 16 (17.6%) of teachers used no violence over two school days (Table 4). Scores for the quality of the classroom environment (emotional support and classroom organisation) and for class-wide child aggression were in the mid-range and scores for class-wide prosocial behaviour were in the low range.

**Table 3:** Teacher and classroom characteristics at pre-test.

**Teacher and classroom characteristics**	**Pre-test data**
Number of children in class [Mean (SD)]	15.26 (6.00)
Number years teaching [Median (IQR)]	14.5 (7.75–22.25)
Number years teaching at this school [Median (IQR)]	8.0 (3–20)
Sex: female [*n* (%)]	90 (98.9%)
Teacher age *n* (%): < 25 25-34 35-44 45-54 55-64 >65	1 (1.1%) 18 (19.8%) 26 (28.6%) 31 (34.1%) 14 (15.4%) 1 (1.1%)
Completed high school [*n* (%)]	79 (86.8%)
Trained teacher [*n* (%)]	32 (35.2%)

**Table 4:** Pre-test and post-test scores for outcome variables and standardised difference.

	**Baseline** ***n =* 91**	**Post-test** ***n =* 91**	**Standardised difference**	***p*-value**
Violence over one school day [Median (IQR)]	6.0 (1.0–17.5)	1.0 (0.0–1.0)	83.33% reduction	< 0.001
No violence over two school days [n (%)]	16 (17.6%)	29 (31.9%)	14.3% increase	0.004
Emotional support [Mean (SD)]	3.70 (0.77)	4.08 (0.65)	0.43 (0.21, 0.65)	< 0.001
Classroom organisation [Mean (SD)]	4.27 (0.80)	4.61 (0.71)	0.43 (0.21, 0.64)	0.002
Class-wide aggression [Mean (SD)]	3.01 (1.43)	3.08 (1.40)	0.09 (−0.12, 0.30)	0.36
Class-wide prosocial behaviour [Mean (SD)]	2.07 (0.79)	2.31 (0.71)	0.26 (0.05, 0.47)	0.02
Depression [Median (IQR)]	12.0 (6.0–20.0)	8.0 (5.0–17.0)	−0.20 (−0.41, 0.01)	0.06

At post-test, there was an 83% reduction in teachers' use of violence across one school day [median (IQR) = 1 (0–1), *p* < 0.001] and a significant increase in the proportion of teachers using no violence over two school days [29 teachers (31.9%), *p* = 0.00] (Table 4). There were significant increases from pre-test to post-test for emotional support [standardised difference (d) = 0.43], classroom organisation (*d* = 0.43), and class-wide child prosocial behaviour (*d* = 0.26). Reductions to teacher depressive symptoms were marginally significant (*d* = −0.20, *p* = 0.06). There was no change in class-wide child aggression (*d* = 0.09, *p* = 0.36).

### Qualitative evaluation

Thirty-seven teachers (one from each school at post-test), participated in the in-depth interviews. There were no refusals. All participants were female, participants had been teaching for a median (IQR) of 15.0 years (8.5–23.5), thirty-three (89.2%) had completed high school, and sixteen (43.2%) had completed a teacher-training qualification. There were no significant differences between teachers who participated in the in-depth interviews and those who were not selected on teacher and classroom characteristics or on pre-test measures of the study outcomes (see Supplementary Table 2).

Teachers who participated in the in-depth interviews also had similar engagement and participation in the intervention as the full sample (see Table 1 for details of full sample). The subsample of teachers attended a mean (SD) of 2.9 (1.0) workshops, participated in a mean (SD) of 7.8 (0.7) in-class support sessions, and completed a mean (SD) of 3.5 (2.8) classroom assignments.

**Figure 1:**
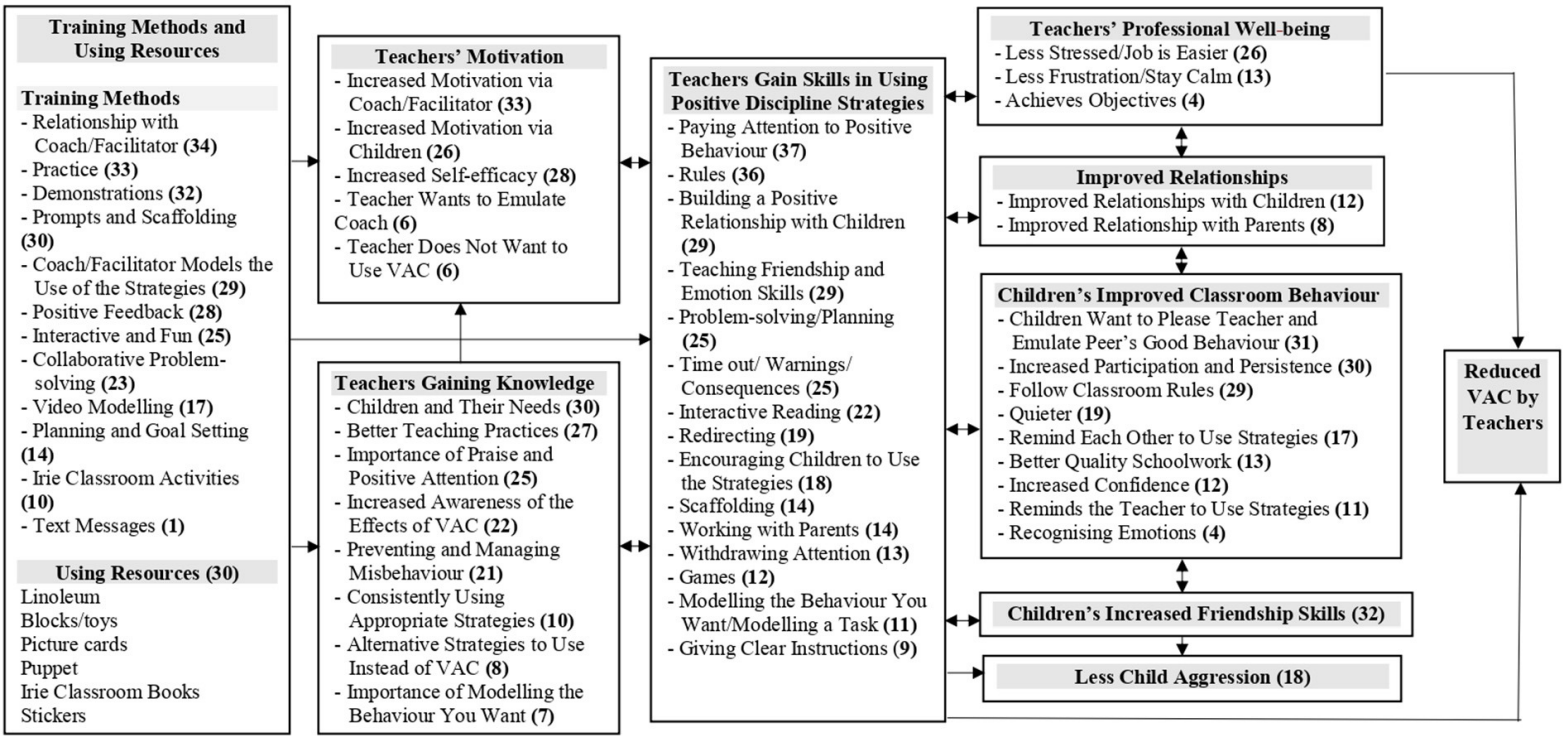
Teachers' reports of the pathways to their reduced use of violence against children (VAC).

**Figure 2:**
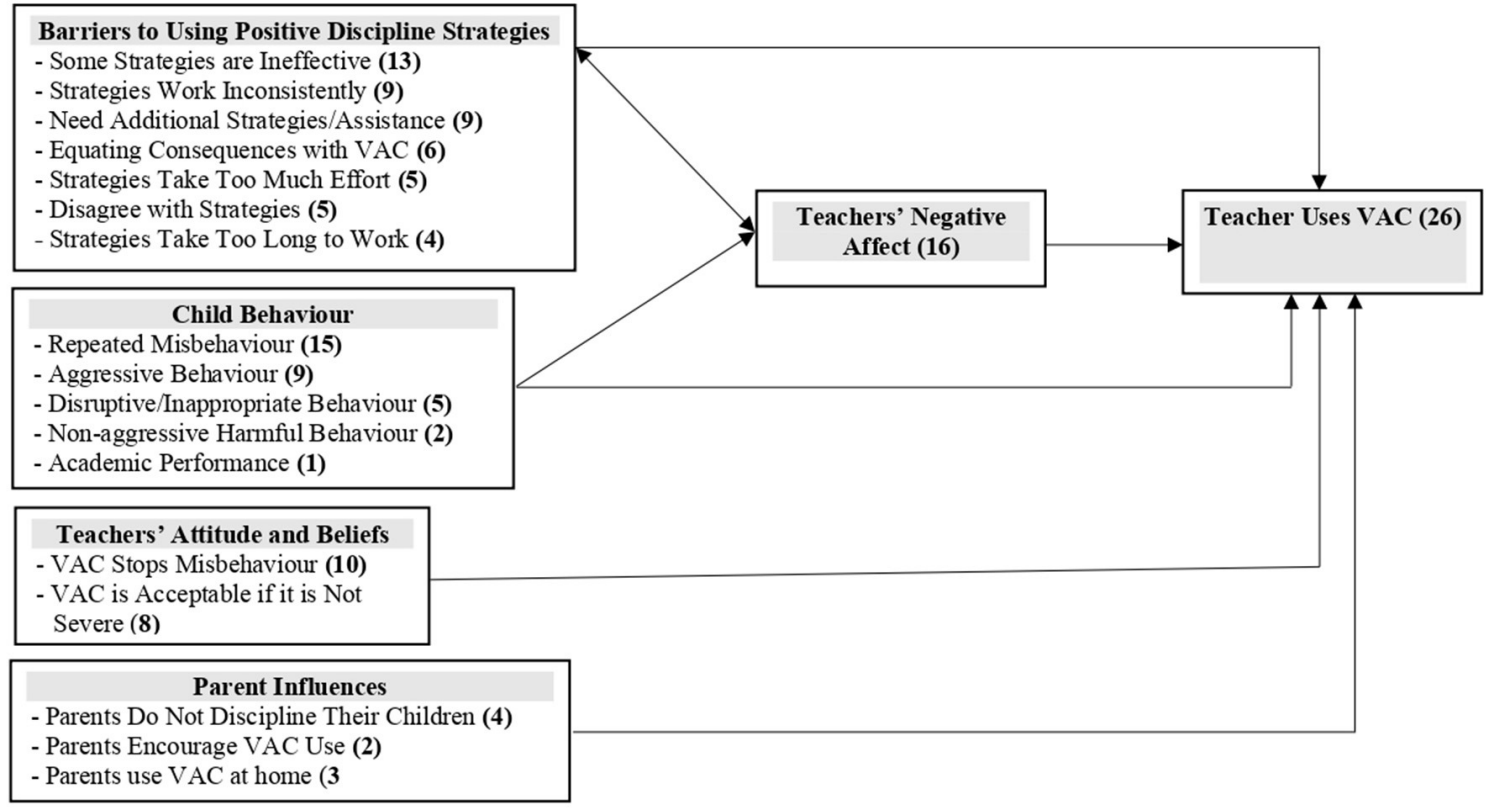
Teachers' reports of why they continued to use violence against children (VAC).

The results of the qualitative evaluation are presented in two main categories: (1) teacher-reported pathways to reduced use of VAC, and (2) teachers' reports of why they continue to use VAC. The pathways are illustrated in Figures 1, 2. In the figures, each box represents a theme and subthemes are listed within each box with the numbers of teachers mentioning each subtheme in parenthesis.

#### Teacher-reported pathways to reduced use of VAC

Teachers reported that the training methods used led to increases in their: (1) motivation to use the strategies, (2) knowledge about child development, appropriate teaching practices and behaviour management, and (3) skills in using the strategies. Teachers reported bidirectional influences between their skills in using the strategies and their motivation and knowledge with motivation and knowledge leading to increased skills and use of the skills leading to further increases in teachers' motivation and knowledge. According to teachers' reports, their use of the strategies led to increased professional wellbeing, improved relationships with children and parents, and improvements in child behaviour including increased friendship skills and reduced child aggression. As teachers recognised the benefits of the programme to themselves (through increased wellbeing and better relationships) and to the children (in terms of improved behaviour), this further increased their use of the positive discipline strategies introduced through the programme. There was evidence of two direct pathways to reduced VAC by teachers: 1) increases in teachers' use of the strategies, and 2) increased teachers' professional wellbeing. See Figure 1 and Table 5 for sample quotes.

**Table 5:** Teachers' reports of factors that led to a reduction in their use of violence against children.

**Training methods and resources** During snack time, she assists the children … if I need any help she would give it. And as she is doing that, she demonstrated some of the strategies. So even while she is helping them, she is also helping me …So that was supportive. (T32) **(Relationship with Coach/Facilitator; Coach/facilitator models the use of the strategies)** She didn't just come and talk and leave it and gone. The fact that she makes me do it in my class, it makes me feel comfortable. And feel like yes, I want to do this with my kids because I realize that they like it so I would want to do it. (T22) **(Practice;** Also **Increased motivation via children)** It was helpful for me because when you were involved in it, you do it hands on, you come back to your class you know exactly what to do. It is not a trial and error, because you did it there [at the workshop] already. (T16) **(Practice)** Maybe you've heard [of] coaching… but then when you go to the workshop they tell you what it is and they show you how to do it, so you know you get a better understanding and try to do it more. (T29) **(Demonstrations)** They were behaving badly and (coach) said, “You praise the ones that are sitting?” So I said “I like how (child) is sitting down,” and (coach) said, “You see it, everybody is sitting down. (T34) **(Prompts and Scaffolding)** Is not beating or shouting going to help children. I learned from the (Irie Tools) book [that] you have to identify what the problem is. Why is the child behaving a certain way? (T30) **(Using Resources, Problem-Solving)**
**Teachers' attitude and motivation** On this particular day, I was talking to this child constantly, getting nothing. Then I started hitting the child, nothing. Then I said but (coach) told me so and so…so let me try it. Then I tried and it worked. Then I said, “Okay. So, I am the one at fault. I need to practice these things.” (T24) **(Increased Motivation via Coach/Facilitator)** Their response, the big smile, and they want to do the work more. So, you want to do that more because if I can get more from you by using this strategy then that's the way to go. (T9) **(Increased Motivation via Children)** I am a little more confident now in terms of how I am able to deliver my lesson, how I am able to get my children involved, how I am able to control my classroom without having to resort to shouting and hitting. (T32) **(Increased Self-Efficacy;** Also **Reduced VAC by teachers)** When I see how she demonstrates and she gets the attention of every student, I feel like I would like that to happen to me too. So we say ‘If she did it, we can do it too.' So all we need to do is practice it and the following day do it. (T6) **(Teacher wants to emulate coach)** I want to stop hitting them because I think that is actually violence as well. (T37) **(Teacher doesn't want to use VAC)**
**Teachers gaining knowledge** The things that we were taught is that children are malleable, and they can learn - it is just for us to take the time.. to sit with a child.. then you can get what you want out of a child. (T37) **(Children and Their Needs)** When she (coach) introduced the games and I implemented them in the lesson, it went smoothly because it made them learn the words quickly… So I learned new ways to teach them certain things. (T33) **(Better Teaching Practices)** Focus on the positive behaviour and not the negative. And if you focus on the negative, you are going to get yourself shouting. But once you say, “Wow I like how (child) is behaving,” everybody behaves. (T34) **(Importance of Praise)** When you slap somebody, it doesn't stop that problem because they are learning that behaviour is what you do. (T17) **(Increased awareness of the effects of VAC)** If you threaten them, they feel discouraged to come to school the next morning. (T31) **(Increased Awareness of the effects of VAC)** When the children are bored, they'll give more trouble. So, when you have transitional songs that help them to move from one activity to another, it makes the transition smoother. (T23) **(Preventing and Managing Misbehaviour)**
**Teachers use new strategies** My classroom became quieter. When somebody shouts, they would even say, “Remember to use your inside voice…” (T4) **(Rules)** They started playing with each other, helping each other and when I start coaching and praising them, they said, ‘Aunty I am sharing with him,. Look, look!' And everybody wants to feel good about themselves now. (T7) **(Paying Attention to Positive behaviour; Children's Increased Friendship Skills; Children Emulate Peers' Good behaviour)** Get to the children level… [if] they are playing a game, you not only let them, you also play along with them. Children like those things. They like when the teachers are involved. (T29) **(Building a Positive Relationship with Children)**
**Teachers' professional well-being** We find out that when we really focus on the behaviours that we want them to display it's easier, things go more smooth and so it's really easier and it helps the children. (T4) **Less stressed/job is easier)** My job as a teacher easier because it helps to manage the classroom in a more efficient way, so I don't have to be come in and being loud and aggressive. (T18) **(Less Stressed/Job is Easier)** I used to shout but now that I got the Irie come in, I am learning how to control those emotions. (T12) **(Stay Calm)** Sometime the children get really out of hand and you just take a deep breath. Then instead of hitting the children, you do something else to get the children's attention. (Teacher 19) **(Stay calm)**
The classroom was less noisy, and the children cooperated more so I achieved more. (T19) **(Achieves Objectives)**
**Improved relationships** What they try to do is emulate each other for positive rewards. So, it has enhanced the classroom environment a bit, it has made managing it a easier. It also takes away the ugliness of the classroom setting and the stress of the teaching-learning environment. (T1) **(Improved Relationship with Children; Teachers' Professional Well-Being)** Sending home Irie Notes, I get parents more interested. Saying, “Very good, Kyra has done her homework,” they want to do it every time because they see that the child is learning more. (T20) **(Improved Relationship with Parents)**
**Children's improved behaviour** Those children who are not working quietly would listen and hear me talking to the ones over there, “Wow, what a lovely coloring you're doing,” and so they would try to go over and start to color too. So, my classroom is much quieter. (T15) **(Children Want to Please Teacher and Emulate Peer's Good behaviour; Less Noise)** There is a child that doesn't like to do any work and when she sees me coaching others, she tries. She sees what they are doing and tries, so I can come along and help her with it. (T29) **(Increased Participation and Persistence; Children Want to Please Teacher and Emulate Peer's Good behaviour)** The praise encourages them to work harder and finish their tasks in time. (T21) **(Increased Participation/Persistence)** I think the children enjoy the rules. We get the children involvement more because for instance when we say, “Eyes on teacher,” everybody would stop and [have] their eyes on teacher. (T6) **(Follow Rules)** I will go out and when I come back, I would find one or two of them modeling me. If they are talking too loud they remind each other, ‘Inside voice'. (T20) **(Children remind each other to use the strategies)** To get all of them who participate, say okay ‘Simon says we are going to do this' then everybody willing now and they get up and start participating. (T3) **(Games, Increased participation)**
**Children's increased friendship skills** I find that when I do the hugging and the praising, they themselves in turn praise each other and hug too. (T16) I also tried the Big Up Cheer… my class loves it…Whenever I do it, I get total class attention because they are listening and they want to try to find ways in which they can compliment their friends. (T1) Once you start coaching them and they start feeling special, you notice they start being friendlier. They start [to] play with each other, they share more with each other more. (T28)
**Less child aggression** When you model, you're like a role play. You form the line and show them what is expected. So, they know that when I said, “In the line,” there is no pushing. They know how to walk instead of run and not to push. (T14) **(**Also **Rules)** …when we encourage them to share, you find that you hear children telling each other that you should share, so rather than fighting for the blocks, they understand [I must give some to that person… (T18) **(**Also **Friendship/Emotions)** Showing the children how to use the friendship skills. We can share. We can switch and swap, things like those. So, we've been using them and the children aren't fighting again. (T27) **(**Also **Teaching Friendship and Emotion Skills)**
**Reduced VAC by teachers** If I usually do four slaps, now I'm trying to do two until I am going down to one then zero, it's stages. (T25) Instead of us running them down to beat them we have certain strategies that we use… (T8) I realize I have been slapping less. We still have children who are aggressive, but [instead] what I do, [is the] naughty corner. (T17)

##### Training methods and relationships

The behaviour change techniques used in the intervention were valued by teachers including the use of demonstration, practice, modeling, positive feedback, prompting, fun, collaborative problem-solving and provision of resources. There was a special salience around the theme of relationships with teachers reporting increased positive relationships between facilitators and teachers, between teachers and children, between teachers and parents, and among the children. Teachers described their workshop-facilitators and in-class coaches as being supportive and they felt comfortable freely sharing the challenges that they experienced in the classrooms. This resulted in the teachers feeling motivated to continue using the strategies, even during difficulties. For example:

“*She motivated me… She was calm even when you say you can't bother, she would just [say] ‘Alright, try this way, try that way.' So, when she is not here you just remember everything. You want to do your job well, so you just try everything…try all the strategies that she taught you*. (Teacher 14) **(Increased motivation via Coach/Facilitator)**.

Teachers reported that their use of the strategies led to improved relationships with the children and their parents. Teachers also reported improved relationships among children in terms of increased friendship skills and less aggression:

“*…when we encourage them to share, you find that you hear children telling each other that you should share, so rather than fighting for the blocks, they understand [that] I can't have it all, I must give some to that person…* (Teacher 18) **(Children's improved friendship skills; Less aggression)**

##### Teachers' motivation and knowledge

Teachers' reported feeling motivated to use the strategies: (1) when they received positive feedback from the facilitator/coach, (2) when the coach explained the rationale for using the strategies, and (3) because they wanted to emulate the coach.

*“…It pushes me more to motivate the children. Because she is praising me, it pushes me to teach them more. If I can feel that way, can you imagine how the children would feel? So that's what I do, I motivate them more. I praise them more.”* (Teacher 27) **(Increased Motivation via Coach/Facilitator)**

They also felt motivated to use the strategies more when they saw the benefits to the children.

*I do it every single day. Because the children love it, they love when you praise them. They smile and they always try to do something more for me to praise them*. (Teacher 32) **(Increased Motivation via Children)**

Most teachers reported increased knowledge related to early childhood education including: (1) understanding of children and their needs, (2) using more supportive and fun teaching practices, (3) the importance of praise and positive attention, (4) how to prevent and manage child misbehaviour and/or (5) the effects of using violence against children. For example, a teacher described the ineffectiveness of corporal punishment in promoting positive behaviour:

*“I realise that when I hit them, I have to be constantly hitting them. But when I start to praise the others, that works better.”* (Teacher 24) **(Increased Awareness of VAC and Its Effects)**

##### Teachers' skills in using positive discipline strategies

All teachers reported increased use of praise and positive attention and nearly all reported teaching and promoting the classroom rules. The other most commonly used strategies were building positive relationships with the children, teaching friendship and emotion skills, problem-solving when difficulties arose in the classroom, use of warnings, time-out and other appropriate consequences for child misbehaviour, and interactive reading. Overall, teachers reported using strategies that promote appropriate behaviour the most, and using strategies to manage misbehaviour less frequently. There was evidence of a direct pathway between use of positive discipline strategies and reduced VAC by teachers:

*“Instead of hitting on a child or shouting at a child, I just use the rules.”* (Teacher 6) **(Rules)**

*“Instead of beating them...I say, ‘I like how (child) is doing this' and then everybody wants to do it.”* (Teacher 26) **(Paying attention to positive behaviour)**

All teachers reported that the use of positive discipline strategies led to improved child behaviour (including increased friendship skills and/or decreased aggression).

*“We get the children's involvement more because for instance when we say eyes on teacher, everybody would stop and their eyes on teacher. When we say use inside voice everybody would whisper instead. And not that loud talking.”* (Teacher 6) **(Rules, Follow classroom rules)**

##### Teachers' professional wellbeing

Teachers reported that a key mechanism to reduced VAC was through their own professional wellbeing, including reduced stress and increased emotional self-regulation. Teachers learnt to stay calm rather than react to children's behaviour in anger, and this helped them to use positive discipline strategies rather than resort to VAC.

*“The training has taught me to pause, I use the word pause because instead of jumping to say something that you shouldn't, you remember and you pause; instead of shouting, you remember and you pause; instead of administering corporal punishment, you remember and you pause, and in these pauses you can think of the strategies that were introduced to you and can figure out in your head which one to use.”* (Teacher 36) **(Stay calm)**

There was evidence of a bidirectional relationship between teachers' use of the positive discipline strategies and teachers' professional wellbeing as utilising the strategies also led to fewer child misbehaviours and reduced teachers' frustration and stress.

*“Before the training programme I would be focusing on the negative behaviour, shouting at that person, calling to that person and wasting a lot of time and draining my energy and so forth.”* (Teacher 29) **(Less stressed/job is easier)**

*“Because the friendship skills cuts down some of the shouting, the fighting, it doesn't frustrate me so much.”* (Teacher 27) **(Teaching friendship skills, Less child aggression, Less frustration)**

#### Teachers' reports of their reasons for continued use of VAC

The majority of teachers (26/37 (70%) reported that they continued to use VAC at times. The main reasons given for continued use of VAC were due to barriers in implementing the positive discipline strategies, as a response to perceived child misbehaviour, and due to poor emotional self-regulation (Figure 2). Less commonly mentioned reasons were due teachers' attitudes and beliefs to VAC and parent influences. See Table 6 for examples of quotes.

**Table 6:** Teachers' reports of reasons for their continued use of violence against children.

**Barriers to using positive discipline strategies** When it's time to pack up [there] is a lot of pushing and fighting. And when you still praise, “Look at that one packing up the toys nicely,” you still have a trouble one that is not doing the correct thing. So, you know you have to raise the stick at that one and then he will follow. (T29) **(Strategies are Ineffective;** also **VAC Stops Misbehaviour)** I don't like [to] see students fighting because they are going to fight a lot and I keep talking to you about it. It is definitely time out or ‘If you slap him again I will slap you.' (T28) **(Equating Consequences with VAC)** I use the clap to rhythm and that worked for a while and it worked in some instances. But as I said because of the distractions from the other class sometimes it just didn't work out at all. (T32) **(Strategies Work Inconsistently)** Give us other suggestions of what to do when they are being disruptive because it really gets frustrating. How to handle a situation and get through to that child without hitting that child. (T19) **(Need Additional Help)** You want the right behaviours now. You are in your class and you want them to learn so you want the behaviour right now. (T2) **(Strategies Take Too Long to Work)**
**Child behaviour** If I warn him three, four times, the fifth time I'm not going to warn him. I'm going to slap him hard (T33) **(Repeated Misbehaviour)** The bad word cursing. The kicking and the biting… when they do that stuff I would give them a little slap in their hands or put them in a little naughty corner. (T26) **(Aggressive behaviour;** Also **Equating Consequences with VAC)** I say, ‘Go and color.' And I give her the paper and she is there searching up my cupboard that she should not be in. So I just have to give her a little slap sometimes. (T9) **(Disruptive/Inappropriate behaviour)** When you carry them out on a field trip and you see that it is very harmful for them to be running wild, not engaging in the line procedure sometimes the human side of you, slaps them… (T20) **(Non-aggressive Harmful behaviour)** If the work is not done as it should. the child says, ‘Yes I understand', but at the end of the day the child go and do the same madness. You say, ‘You need two slap man.' (T24) **(Academic Performance)**
**Teachers' attitudes and beliefs** You are praising them, and they are still doing that little rude thing and you will say to them, ‘Listen. If you don't stop doing that, I am going to slap you.' And hearing that from you, they stop, they don't want you to slap them. (T5) **(VAC Stops Misbehaviour)** Some students work when they get the slap. (T26) **(VAC Stops Misbehaviour)** Slapping them is just in their hand middle. It is not really harming them or anything. (T14) (**VAC is Acceptable**)
**Parent influences** It would be nice if you could help the parents so that they know how to treat their children so that when they come to school they don't have to be so rude that the teachers have to slap them. (T14) **(Parents Do Not Discipline Children)** They say, “Teacher, as long as you don't hit him in his eye.” And I turn up their bottom and I slap them on it. (T33) **(Parents Encourage VAC)** Some of the babies are just stubborn and at home them use to the boofing (beating). (T29) **(Parents Use VAC at Home)**
**Teachers' negative affect** …When it's lesson time, he will make some funny sounds and he will be staring at me in the eyes and you will try all kinds of things…I'll try the behaviour plan with him…and I'll be there teaching the lesson…and I would be looking at him and he will continue and I will try the strategies. And he will continue to be rude and he will get up out of his seat and run to the other side and hit somebody and sometimes it feels overwhelming that you are teaching and I just want to give him a slap. (T22) **(**Also **Strategies are Ineffective, Repeated Misbehaviour)**
**Teachers' use of VAC** Sometimes I do give them a little touch. (T10) You give them a little touch but you don't beat like you are at home, no. You may just hold the little hand and say I expect from you, yuh know and they respond. You know, you not going to batter up the children. (T16) I will tap them. (T21) Just a little pat. (T28)

##### Barriers to using positive discipline strategies

Teachers reported several barriers to their consistent use of the strategies in the classroom. Some strategies were perceived to be ineffective, to work inconsistently, to take too much effort and/or take too long to work in practice. These barriers to strategy use were sometimes related to the context in which the teachers worked. Some teachers reported that larger class sizes and/or insufficient space and resources made it difficult for them to consistently use the strategies.

*You want the right behaviours now… but some strategies (now just have to..) just take time. The most I had this week is 19 - I find that I can use the strategies with this number of children, but when me have 27 pikney (children) in front of me oh my gosh you going to say things, you don't want it to come out*. (Teacher 2) **(Strategies take too long to work)**

In addition, some teachers of three-year-old children reported that strategies involving teaching rules, friendship and emotion skills were less effective and/or difficult to use with the younger children; while a teacher of the 5–6-year-old children (who were transitioning to primary school), disagreed with some strategies as she believed that the focus on positive behaviour and praise wouldn't prepare children for the primary school classroom.

*“You have to remember that they are small but at the same time you have to teach them that they are going into a different world (primary school) where they're not going to get that (praise).”* (Teacher 33) **(Disagree with strategy)**

There was also some evidence that teachers equated non-violent consequences with VAC use in that they considered either strategy to be appropriate for certain behaviours and didn't appear to differentiate between them:

*“You spoke to her once and she do it again and the third, so 3 strikes and you are out and so she always let the three strikes catch her so she have to get a little pat or she go in the time-out corner.”* (Teacher 9) **(Equating consequences with VAC)**

##### Child behaviour

Teachers reported using VAC for child misbehaviours that they perceived to be particularly severe, especially repeated misbehaviour and aggressive behaviour.

*“You only clap them when they harm another person or they are trying to harm themselves.”* (Teacher 14) **(Aggressive behaviour)**

*“If I'm teaching the class and the child is being disruptive and I speak to the child and the child continues, I will give the child a slap or two.”* (Teacher 19) **(Repeated misbehaviour)**

##### Poor emotional self-regulation

Another important pathway to continued VAC by teachers was due to teachers' negative affect (e.g., frustration and anger). When teachers were frustrated by children's behaviour, they were more likely to resort to VAC.

*“You know when you see certain behaviour and you just feel upset and all that you just want to knock the child.”* (Teacher 15) **(Teachers' negative affect)**

Others reported feeling frustrated when they used the strategies, and they are ineffective or took too long to correct the behaviour.

*“If I keep talking to a child for one particular incident over and over I tend to get irritated at some point or another and as I said I would hold the child and physically, ‘Sit! I said you are to sit.”'* (Teacher 1) **Teachers' negative affect)**

##### Teachers' attitudes and beliefs and parent influences

A minority of teachers reported a direct link between their attitudes to VAC and VAC use. For example, some teachers reported that VAC was an effective method of managing child misbehaviour and some teachers believed that VAC was an acceptable form of punishment as long as it didn't lead to severe physical abuse:

*“I remember when I was going to school and I get, is not slap I get. They beat you. A tap, right. There is nothing wrong with that. When you take up a belt and beat a child, that is where something is wrong.”* (Teacher 21) **(VAC is acceptable if it is not severe)**

Parent influences were mentioned by a small minority of teachers. This included: 1) parents not disciplining children at home, leading to severe child misbehaviour at school, 2) parents supporting VAC use by teachers, and 3) parents' use of VAC at home justifying teachers' use of VAC at school.

*“I will ask them, ‘Your parents slap you at home?' It's like something they are used to. So, if the teacher slaps you, it's like nothing.”* (Teacher 14) **(Parents use VAC at home)**

## Discussion

In a pre-post evaluation, the Irie Classroom Toolbox reduced VAC by teachers by 83% and increased the proportion of teachers using no violence by 14%. However, 68% of teachers were observed to use VAC at least once over 2 days of observation at post-test. These findings were corroborated through the qualitative evaluation with all teachers reporting reduced VAC, but 70% reporting continuing to use VAC at times. The reductions in teachers' use of VAC were accompanied by significant benefits to the observed quality of the classroom environment and class-wide prosocial behaviour, although no benefits were found for class-wide child aggression. Reductions were also found for teachers' depressive symptoms. Teachers reported that the behaviour change techniques used in the intervention led to increased motivation, knowledge and skills which in turn led to improved child behaviour, improved relationships and improved professional wellbeing. There was evidence of bidirectional influences with improved child behaviour, relationships and professional wellbeing also leading to increased use of positive discipline skills by teachers which in turn increased teachers' motivation and knowledge. Teachers reported that the direct mechanisms to reduced VAC were through their increased use of positive discipline strategies and improved professional wellbeing. The main reasons for teachers' continued use of VAC were due to barriers faced in using the positive discipline strategies, teachers negative affect and certain child behaviours, especially child repeated misbehaviour and child aggression. Attitudes to violence and parental influences were also mentioned as reasons for continued use of VAC by a minority of teachers.

The findings from this mixed method evaluation are useful for informing revisions to the content of the programme to strengthen its effectiveness in reducing violence against children by teachers (see Table 7). For example, the in-depth interviews highlight the importance of training in alternative discipline and emotional self-regulation as these are key factors in the pathways to reduced VAC and are also reasons given for teachers' continued use of VAC. These factors have been recognised as core components of effective violence-prevention parenting programmes (29), and were also described as the most salient mechanism to reduced VAC by parents who participated in the Irie Homes Toolbox (24). Previous qualitative evaluations of violence-prevention programmes in primary schools in LMIC have also reported that training in alternative discipline strategies is a key mechanism to reduced VAC by teachers (22, 30) with emotional regulation (22), improved relationships (22, 30) and improved child behaviour (22) also being described as being on the pathway of change. An important finding was that some teachers understood corporal punishment to be more severe child abuse and they did not differentiate between appropriate consequences (such as time-out) and slapping a child. In Uganda, teachers and students expressed a similar belief that VAC is acceptable if it is proportionate and fair (30). Addressing teachers' knowledge, attitudes and beliefs related to VAC may be one strategy for further reducing VAC by teachers.

**Table 7:** Using the results of the mixed method assessment to inform the further scale-up of the Irie Classroom Toolbox.

**Factors related to programme content**	
**findings**	**Suggested actions**
•Although large reductions to teachers' use of violence against children (VAC) were found, the majority of teachers continued to use VAC.	•There is a need to identify strategies to eliminate teachers' use of VAC. Suggestions are given below.
•One of the main reasons for teachers' continued use of VAC was due to barriers faced when using appropriate classroom behaviour management strategies.	•Provide more support for problem-solving how to deal with difficult situations. For example: 1) include more role plays and practice activities in workshops and 2) discuss and reinforce appropriate expectations of young children. Teacher may also require ongoing support after the end of programme implementation to fully adopt the practices.
•Another commonly mentioned reason for teachers' continued use of VAC was due to teachers' poor emotional self-regulation skills.	•Include a greater focus on promoting teachers' emotional self-regulation and executive function skills including teaching calm-down techniques and increasing support for goal setting, planning and problem-solving.
•Teachers reported difficulties in managing certain child behaviours, especially repeated misbehaviour and aggressive acts.	•Provide more support and advice on how to manage more severe child behaviours including role play and rehearsal in workshops and increased support with individual behaviour planning for children with behaviour problems.
•We found no benefits to observed class-wide child aggression in this study or in our previous evaluation of the Irie Classroom Toolbox.	•Design additional materials to help teachers to manage children's aggressive behaviour. This will include encouraging teachers to respond consistently to child aggression and continuing to teach friendship and emotion skills.
•Teachers' beliefs and attitudes related to the use of corporal punishment were another reason reported by teachers for their continued use of VAC.	•Include content to explicitly challenge teachers' attitudes to violence against children including the short and long term negative effects of using VAC and positive effects of using positive discipline strategies.
•Teachers' used terms such as ‘touch', ‘brush off', ‘tap' to describe ‘milder' forms of corporal punishment and they did not view these as violence against children.	•Provide clear definitions of violence against children, including corporal punishment and psychological aggression.
•Teachers' reports indicated a perceived equivalence between appropriate consequences (e.g. time-out) and use of violence.	•Include more activities to help teachers understand the concept of appropriate consequences including logical and natural consequences, and how these differ from violence against children.
**Factors related to programme implementation**	
**findings**	**Suggested actions**
•Larger reductions to teachers' use of VAC were found in this evaluation compared to the previous round (previous round, median number of VAC: 7 at pre-test, 3 at post-test; current study, median: 6 at pre-test, 1 at post-test).	•Facilitators had received more training and supervision and were more experienced which may explain this finding. It is important to advocate for a sufficient duration and frequency of training and supervision for the government staff who will be implementing the programme as it is scaled up. Promoting staff retention is also important.
•The behaviour change techniques used in programme delivery were reported to be important in changing teachers attitudes, knowledge and skills.	•As the programme is scaled up, it is important that programme facilitators are given high quality training and support in the use of, and rationale for, the behaviour change techniques used to deliver the training (e.g. demonstration, rehearsal and practice, modeling, giving positive, constructive feedback).
•The quality of relationships between the facilitators and teacher, teachers and children, and teachers and parents were a theme running through the evaluation.	•All stakeholders need to understand the importance of positive relationships for quality implementation and need the skills to develop and maintain these supportive relationships (e.g. reflective listening, collaborative problem-solving).
**Factors related to programme monitoring**	
**findings**	**Suggested actions**
•Teacher engagement, participation and satisfaction was high when the programme was implemented by staff hired by the research team and it is likely that these are key factors for programme effectiveness.	•It is important to promote the use of monitoring tools to measure teacher engagement, participation and satisfaction as the programme is scaled up and to use the results to ensure high levels are maintained.
•The use of collaborative, participatory and fun training methods and evidence-based behaviour change techniques are important in programme delivery.	•Programme supervisors need monitoring tools and appropriate training in evaluating facilitators' skills in delivering the programme and providing additional support when necessary. Facilitators also need to be encouraged to use self-evaluation tools to reflect on their own skills in programme delivery and identify areas for improvement.
•The quantitative evaluation showed that the intervention was effective in reducing teachers' use of VAC, increasing the quality of the classroom environment in terms of teacher practices and child prosocial behaviour.	•It is important to continue to monitor effectiveness as the programme is scaled up. Assessments of teachers' classroom management skills and child behaviour can be incorporated into the existing government inspections using simple to use checklists adapted from the outcome measurements used in our research.

The Irie Classroom Toolbox has been designed to be integrated into the early childhood educational network in Jamaica with training and supervision to be provided by government early childhood officers as part of their routine duties. The findings from our MEL activities give insights into teachers' preferred training methods and the mechanisms of action of the intervention, thus providing important guidance related to programme implementation and programme monitoring as it is scaled up in Jamaica (see Table 7). This includes the importance of using evidence-based behaviour change techniques, fun and interactive training methods and building supportive relationships between facilitators and participants. Qualitative evaluations of early childhood parenting programmes in LMIC have highlighted the importance of these techniques in promoting engagement and learning (31–33) and there is growing empirical evidence of their importance for participant engagement [(34); Bernal et al.]^2^ and programme effectiveness (34, 35) (see footnote 2). In addition, we reported larger reductions to teachers' use of VAC in this round of implementation compared to our previous round (see Table 7) (17). The main difference in implementation was the fact that the programme staff were more experienced and had received more training and supervision. This highlights the importance of providing sufficient training and ongoing supervision to programme staff as learning to utilise effective training methodologies requires practice with skills developing over time (34, 36). As the programme is scaled up, it will also be important to continue to monitor intervention implementation including: (1) teacher satisfaction and engagement with the intervention, (2) facilitators' skills in implementing the intervention, and (3) the effectiveness of the intervention on teacher and child outcomes (Table 7).

Our MEL activities also point to the limitations of teacher-training alone for eliminating VAC at school. Corporal punishment is banned by law in Jamaican early childhood institutions and yet as seen in this study, VAC continues to be widely used. Reviews of the global status of VAC in schools indicate that this situation is common across many countries with legal bans (37, 38). In Jamaica, education and training alone was insufficient for ensuring teachers' compliance with the law against corporal punishment and monitoring and enforcing compliance is also necessary. In addition, interventions to change attitudes and beliefs relating to VAC within the wider community may be necessary. Implementing complementary teacher and parent, early childhood violence prevention programmes is one step in this process to ensure a shared understanding and co-ordinated approach to positive discipline at home and at school. Conducting violence-prevention programmes in primary and secondary schools with the aim of preventing VAC by teachers in all educational institutions could also help change societal attitudes toward VAC at school. Additionally, mass media campaigns may be helpful for awareness raising and behaviour change at the population level (39, 40).

This study has demonstrated the value of MEL activities to inform future implementation of an early childhood, violence prevention, teacher-training programme. The study illustrates four of the five concepts described in the Measurement for Change framework. The MEL activities were informative and dynamic in that information was gained from quantitative and qualitative methods and this information was used to guide future decision-making related to the content, process of delivery and future monitoring of the intervention as it is implemented at scale. We found evidence of the importance of MEL activities being interactive from the salience of the theme of relationships in the qualitative data. Teachers reported that the positive, supportive relationships they had with the facilitator were mirrored in their relationships with children and parents and in more friendly behaviours among the children. Including methods for monitoring relationships will be an important component of the MEL process as the programme is scaled-up. A further illustration of the interactive concept is given in the evidence of bidirectional effects (see Figure 1). For example, teachers use of positive discipline strategies led to improved child behaviour and improved child behaviour encouraged teachers to use the strategies more. Finally, the data from the MEL activities illustrate the importance of being people-centered. Different teachers faced different challenges and expressed different needs related to implementing the intervention. Although the content of the Irie Classroom Toolbox is relevant for all teachers, how this content is operationalised in each teachers' individual classroom context will differ. Furthermore, additional support may be required at times, for example, in emotional self-regulation skills, in dealing with specific child behaviours, and/or in changing norms and attitudes to VAC. In this study, our MEL activities were not inclusive as due to resource constraints, we were only able to collect data from teachers and classrooms, and not from other relevant stakeholders.

The strengths of the study include the mixed-method approach that included quantitative and qualitative data. The qualitative data triangulated the results of the quantitative data and provided valuable information on the perspectives of teachers relating to the mechanisms of action of the intervention and reasons for continued VAC use. Incorporating participant perspectives into MEL activities is an essential component of the Measurement for Change Approach (25). Measurements were conducted by persons masked to the study design and most had good psychometric properties, with the exception of class-wide child prosocial behaviour which had low stability. Although, 16% of teachers were lost at post-test, there was no differences between those lost and found on any of the characteristics measured at pre-test. In addition, we randomly selected one teacher from each school to participate in the in-depth interviews and the selected teachers were not significantly different from those not selected on pre-test characteristics and had similar levels of engagement and satisfaction with the intervention. The views of the teachers who participated in the in-depth interviews are thus likely to be reasonably representative of the wider sample. The measurements of teachers' use of VAC were through independent observation, thus reducing the bias of teacher reports which tend to underestimate VAC use (40, 41). The young age of the children prevented the use of child-reported measures. It is possible that teachers behaved differently during the observational assessment. However, the presence of the observer is generally non-intrusive in these preschool classrooms given the structural conditions, with high noise levels and several classrooms sharing a common space. In addition, there is evidence that when observations conducted over a whole school day, the effects of an observer on teacher behaviour are reduced (42) and in our trial we found reductions in teachers' use of VAC did not differ across the school day (17).

The limitations of the study are that this was a pre-post study with no untreated control group. Furthermore, as the preschools were in the wait-list control group of a cluster-randomised trial, they had participated in multiple rounds of measurement which may have resulted in changes to teachers' behaviour. Only thirty-seven teachers (one per preschool) participated in the in-depth interviews and hence the reported mechanisms of action require cautious interpretation and need to be investigated in future empirical research. Social desirability bias may also have influenced teachers' responses during these interviews as the teachers were aware that the interview was being conducted on behalf of the Irie Toolbox Team. All respondents were female which reflects the lack of male early childhood teachers in Jamaica (only 1/91 teacher (1.1%) in the study preschools was male). It is possible that male teachers may experience the intervention differently. We conducted the in-depth interviews at the end of the intervention only and hence we were unable to track teachers' perceptions of the mechanisms of change throughout the intervention implementation and we did not have the resources to get feedback from teachers on the interpretation of the results. In addition, in this study, we only report data from teachers and classrooms. Other important stakeholders include parents, government field officers and their supervisors, members of the school board, and members of the local communities. It will be important to include the perspectives of a wider group of stakeholders in future studies. Finally, all participating preschools were situated in urban areas and future studies need to include preschools from rural and semi-rural areas of Jamaica.

## Conclusion

In this study, we demonstrate how embedding MEL activities into ongoing intervention implementation can help to plan for implementation at scale. We used a mixed-method evaluation of the Irie Classroom Toolbox when implemented with teachers in preschools from the wait-list control group of a cluster-randomised trial. We had previously demonstrated that the Toolbox led to large reductions in teachers' use of VAC, although the majority of teachers continued to use VAC at times (17). The MEL activities in this round of implementation confirmed these findings and provided insights into teachers' perspectives of the mechanism of action of the intervention and their reasons for continuing to use VAC. The information is useful for preparing additional content to further reduce and ultimately eliminate VAC by teachers. In addition to strengthening the intervention, the MEL activities also provide valuable information to guide the process of scaling the intervention, including provision of high-quality training, supervision and ongoing monitoring and evaluation.

## Data availability statement

The raw data supporting the conclusions of this article will be made available by the authors, without undue reservation.

## Ethics statement

The studies involving human participants were reviewed and approved by School of Psychology, Bangor University Ethics Committee and University of the West Indies Ethics Committee. The participants provided their written informed consent to participate in this study.

## Author contributions

HB-H contributed to funding acquisition. MB and TF contributed to project investigation and data curation. MB and HB-H contributed to data analysis. MB wrote the original draft. HB-H and TF reviewed and edited the manuscript. All authors contributed to the conceptualization of the study and project administration.

## Footnotes

^1^Baker-Henningham H, Bowers M, Francis T. The process of scaling early childhood violence prevention programs in Jamaica. Special issue on promoting early childhood globally through caregiving interventions. *Pediatrics* (submitted).

^2^Bernal R, Gomez ML, Perez-Cardona S, Baker-Henningham H. Quality of implementation of a parenting program in Colombia and its effect on child development and parental investment. *Pediatrics* (submitted).
