# Supplementary material for: The Irie Classroom Toolbox: Mixed method assessment to inform future implementation and scale-up of an early childhood, teacher-training, violence-prevention programme
Source: Front Public Health. 2022 Dec 13;10:1040952. doi: 10.3389/fpubh.2022.1040952 (PMC9792689; doi:10.3389/fpubh.2022.1040952)
Supplement: Supplementary file 1 [file Data_Sheet_1.PDF]

Supplementary Tables

Supplementary table 1. Classroom and teacher characteristics by loss at post-test

	**Found n=91**	**Lost n=17**	**p-value**
Number of children	15.26 (6.00)	15.00 (5.17)	0.87
Number years teaching	14.5 (7.75-22.25)	9.00 (3.88-19.00)	0.20
Number years teaching at this school	*8 (3-20)*	*1 (0.6-10)*	*0.31*
Sex: female	90 (98.9%)	17 (100%)	0.66
Teacher age:			0.13
<25	1 (1.1%)	1 (5.9%)	
25-34	18 (19.8%)	4 (23.5%)	
35-44	26 (28.6%)	8 (47.1%)	
45-54	31 (34.1%)	2 (11.8%)	
55-64	14 (15.4%)	1 (5.9%)	
≥65	1 (1.1%)	1 (5.9%)	
Completed high school	79 (86.8%)	12 (70.6%)	0.18
Trained teacher	32 (35.6%)	6 (35.3%)	0.98
Violence over one school day	6.0 (1.0-17.5)	9.0 (2.0-16.5)	0.87
Emotional support	3.70 (0.77)	3.54 (0.60)	0.43
Classroom organisation	4.27 (0.80)	4.03 (0.60)	0.25
Classwide aggression	3.01 (1.43)	2.99 (1.61)	0.96
Classwide prosocial behaviour	2.07 (0.79)	2.07 (0.62)	0.98
Depression	12 (5.75-20.25)	17 (10.0-24.5)	0.24
No violence over 2 school days	16 (17.6%)	2 (11.8%)	0.56

Supplementary table 2. Classroom and teacher characteristics by participation in qualitative evaluation

	**Selected to participate in in-depth Interviews** n=37	**Not selected to participate in in-depth interviews** n=54	**p-value**
Number of children	15.03 (6.89)	15.42 (5.37)	0.77
Number years teaching	15 (8.5-23.5)	13 (6-22)	0.20
Number years teaching at this school	*9 (3-21)*	*7 (2.5-19)*	*0.55*
Sex: female	37 (100%)	53 (98.1%)	0.41
Teacher age:			0.40
<25	1 (2.7%)	0 (0%)	
25-34	5 (13.5%)	13 (24.1%)	
35-44	10 (27.0%)	16 (29.6%)	
45-54	15 (40.5%)	16 (29.6%)	
55-64	5 (13.5%)	9 (16.7%)	
≥65	1 (2.7%)	0 (0%)	
Completed high school	33 (89.2%)	46 (85.2%)	0.73
Trained teacher	16 (43.2%)	16 (29.6%)	0.18
Violence over one school day	6.0 (1.25-18.25)	6.0 (1.0-16.5)	0.83
Emotional support	3.78 (0.68)	3.65 (0.83)	0.45
Classroom organisation	4.26 (0.79)	4.27 (0.79)	0.98
Classwide aggression	3.08 (1.21)	2.96 (1.58)	0.69
Classwide prosocial behaviour	2.17 (0.73)	1.99 (0.82)	0.29
Depression	12 (5.5-19.0)	11.5 (5.75-21.25)	0.94
No violence over 2 school days	6 (16.2%)	10 (18.5%)	0.78
