# Supplementary material for: The Irie Classroom Toolbox: Mixed method assessment to inform future implementation and scale-up of an early childhood, teacher-training, violence-prevention programme
Source: Front Public Health. 2022 Dec 13;10:1040952. doi: 10.3389/fpubh.2022.1040952 (PMC9792689; doi:10.3389/fpubh.2022.1040952)
Supplement: Supplementary file 2 [file Data_Sheet_2.PDF]

Supplementary Material. The Irie Classroom Toolbox: Mixed Method Assessment to Inform Future Implementation and Scale-up of an Early Childhood, Teacher-Training, Violence-Prevention Programme

Coding Sheet for Observations of Teachers' Use of Violence Against Children (VAC)

School ID: ___________ **Teacher ID:** ____________ **Date:** __________ **Observer:** __________ **# Students**: _______

Time of day: Early morning Late morning Afternoon **Duration:** __________ (minutes)

				TOTAL
**Physical**				
**Verbal**	Name calling			
	Verbal threats			
	Rejection of child			
	Encourage children to reject/harm other children			
**Other**				

Time of day: Early morning Late morning Afternoon **Duration:** ___________ (minutes)

				TOTAL
**Physical**				
**Verbal**	Name calling			
	Verbal threats			
	Rejection of child			
	Encourage children to reject/harm other children			
**Other**				

School ID: ___________ **Teacher ID:** ____________ **Date:** __________ **Observer:** __________ **#Students**: ________

Time of day: Early morning Late morning Afternoon **Duration:** ___________ (minutes)

				**TOTAL**
**Physical**				
**Verbal**	Name calling			
	Verbal threats			
	Rejection of child			
	Encourage children to reject/harm other children			
**Other**				

			**TOTAL FOR THE DAY**
**Physical**			
**Verbal**	Name calling		
	Verbal threats		
	Rejection of child		
	Encourage children to harm others		
**Other**			
**TOTAL VAC**			

Definitions

PHYSICAL NEGATIVE
Any action that deliberately inflicts pain or intends to cause pain to the child. The touch must be rough. Examples include hitting (with hand or with an object), forcefully pushing or pulling a child, pinching, poking, shaking and making child stand in an uncomfortable position (e.g. standing with hands outstretched).

VERBAL NEGATIVE
Includes name calling, verbal threat, rejecting a child and encouraging children to harm, insult or exclude another child.

<u>Name calling</u>
Referring to or calling child a name that is emotionally abusive or insulting and obviously derogatory. The name used labels the child as a whole and not the behavior he/she displays or the work he/she has done.

<u>Verbal Threats</u>
Teacher states intention to take hostile action against child. The action is frightening and/or one that would cause the child physical pain. This includes threats to hit or harm, threats that frighten the child and threats of inappropriate consequences e.g. forcing child to be hungry by taking away their lunch. The teacher must explicitly state what she is going to do. If the threat is said as a question (e.g. "*Do you want me to (verbal threat)?"),* code it.
Verbal threat is not related to the tone of the teacher, that is, whether the teacher uses a harsh or pleasant tone is irrelevant. Code the words said.

<u>Rejecting a Child</u>
The teacher rejects the child as a whole (not to be confused with rejection of work done).

<u>Encourage children to harm, insult or exclude another child.</u>
Teacher encourages a child to harm another child, whether in retaliation or otherwise. Teacher may also encourage other children to insult or exclude a child.

OTHER NEGATIVE
Methods of intimidation/ scare tactics. This includes non-verbal threats (e.g. raising ruler as though to strike child), shouting loudly in child's face and banging a stick hard on desk in front of child.

<u>How to code</u>
Each instance of abuse is coded as a single tally.
When more than one abusive action is occurring at the same time, a tally should be given for each act. For example, if the teacher is hitting a boy while name calling, a tally is given for each hit and one for every name she calls him.
E.g. Teacher: you stupid boy! Roughly pulls boy then slaps him.
Code 3 tallies: 1 verbal (name calling) and 2 physical (rough pull and slap).

When coding, ***<u>do not judge the reaction of the child or what is happening in the classroom</u>***, code according to the actions of the teachers.

Observers should start coding when school begins and continue for the entire day except during break and lunch time. Rest time is also coded. There are three observation periods: before break, after break and after lunch. Use a separate box for each observation period.

Full details with examples and non-examples of each category of behaviour are given in an observation manual. For ethical reasons, this manual is not included.
